# Supplementary material for: Sex peptide receptor-regulated polyandry modulates the balance of pre- and post-copulatory sexual selection in Drosophila
Source: Nat Commun. 2019 Jan 17;10:283. doi: 10.1038/s41467-018-08113-w (PMC6336784; doi:10.1038/s41467-018-08113-w)
Supplement: Supplementary file 1 — Supplementary Information [file 41467_2018_8113_MOESM1_ESM.pdf]

Supplementary Information

Sex peptide receptor-regulated polyandry modulates the balance of pre- and post-copulatory sexual selection in *Drosophila*

Morimoto et al.

Supplementary Figures

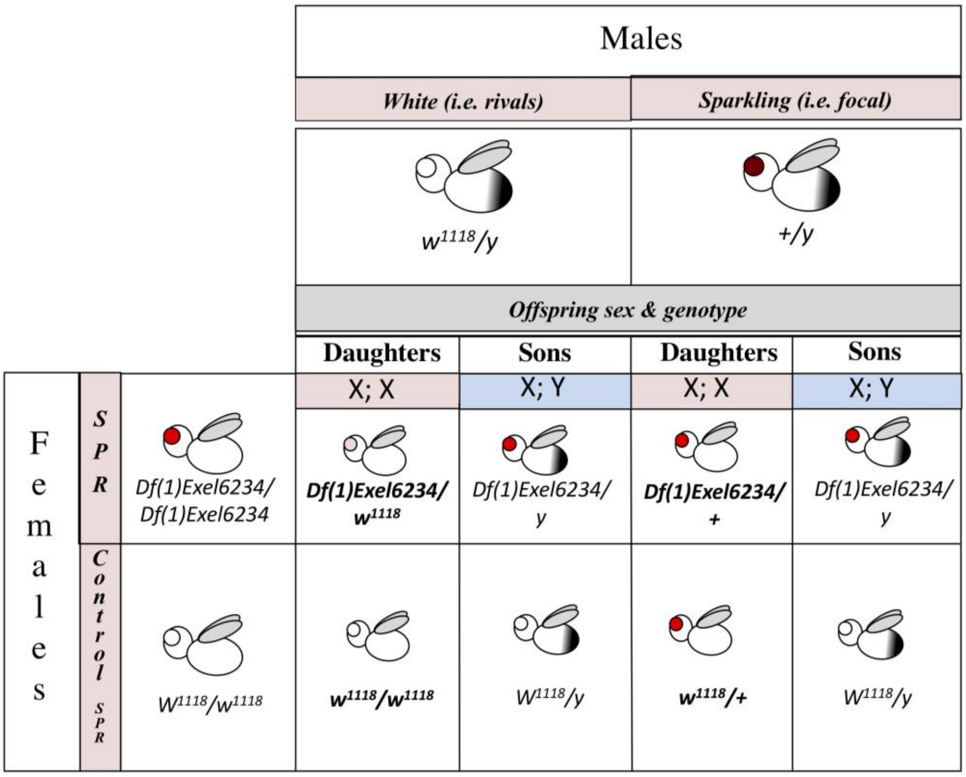

Supplementary Figure 1. Fly husbandry and paternity share of the focal male in the SPR experiment. Competitor males and all females (including *SPR*) treatments were homozygous for the recessive w^{1118} mutation (i.e. they did not bear the wild-type *white* allele) located on the X chromosome, which gives white eyes. *SPR*- treatment females carried a genetic deficiency, *Df(1)Exel6234*, also located on the X chromosome, covering the *sex-peptide receptor* and 4 other genes of unknown function [1]. *Df(1)Exel6234* includes an insertion of a *white+* transgene that partially rescues the w^{1118} phenotype: heterozygote *Df(1)Exel6234* carrier females in a w^{1118} background have orange eyes and male carriers have red eyes. Focal males in all treatments possessed the wild-type dominant *white* gene (which gives red eyes) on the X chromosome, and were also homozygous for the recessive *sparkling*^{poliert} mutation (*spa*) on the 4th chromosome. The female offspring of focal males therefore inherited an X-chromosome bearing the wild-type *white* gene from their father, and displayed wild-type red eyes, whereas the daughters of rival (w^{1118}) males were white-eyed in the controls, or orange-eyed in the *SPR* treatment. Note that it is only possible to assign paternity for the focal male through the eye phenotype of daughters. Sons could not be differentiated because they received the focal male's Y chromosome, not the wild-type *white*-bearing X chromosome. Note, only 1st chromosome (X and Y) genetics are shown here.

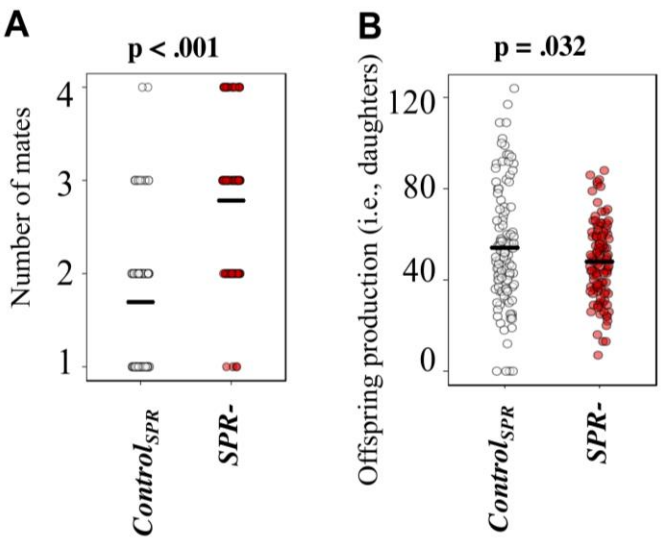

Supplementary Figure 2. Female SPR- mating and offspring. **a.** The number of mates of females in the *SPR*- experiment. **b.** Offspring (i.e. daughter) production of females in the *SPR*- experiment. *N* = 108 for control$_{SPR}$ and *N* = 111 for *SPR*- treatment. Horizontal black line represents the mean of the data.

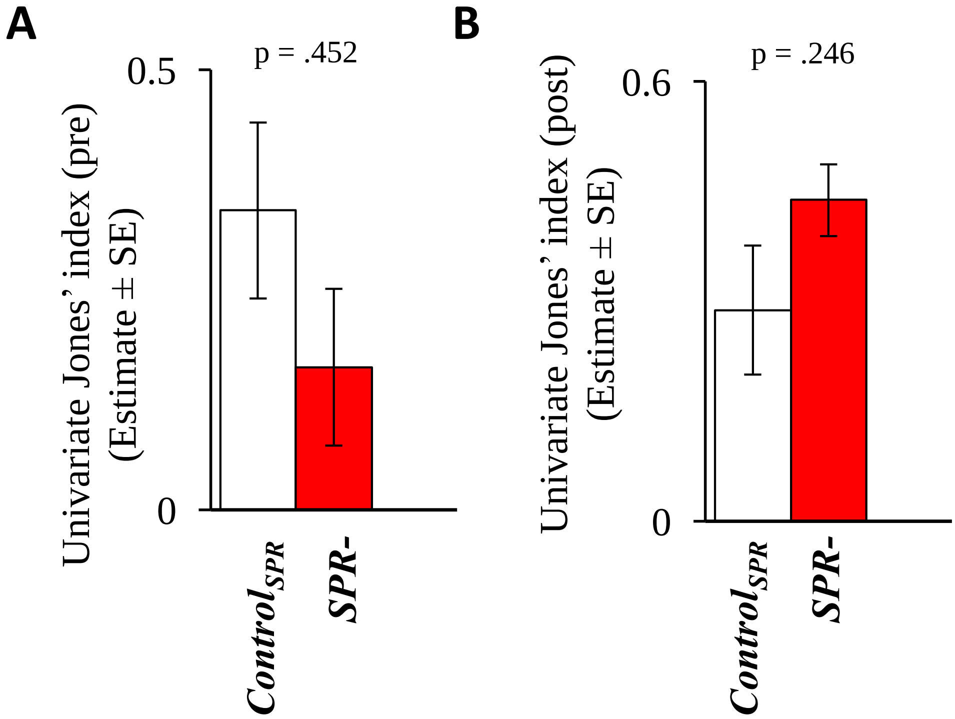

Supplementary Figure 3. The univariate pre- and post-copulatory Jones' Indexes in the *SPR*- experiment. **a**. The univariate pre-copulatory Jones' index. **b.** The univariate post-copulatory Jones' index. *N* = 27 for the control$_{SPR}$ and *N* = 29 for the *SPR*- treatment. Error bars refer to the standard error of the mean (SE).

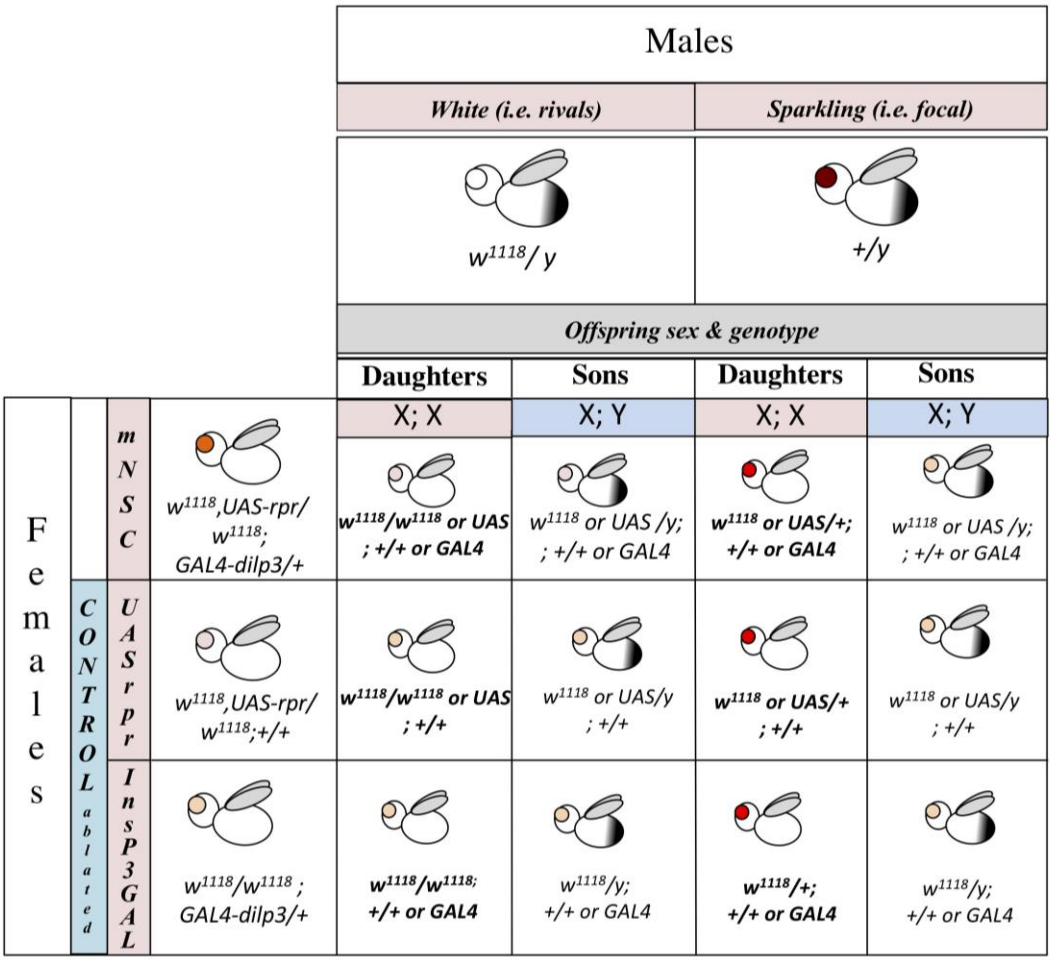

Supplementary Figure 4. Fly husbandry and paternity share of the focal male in the mNSC-ablated experiment. As in the *SPR* experiment, competitor males were homozygous for the recessive w^{1118} mutation (i.e. they did not contain the wild-type *white* gene) located on the X chromosome, which conferred them the white-eye phenotype. All females - including UAS-*rpr* and GAL4-dilp3 heterozygotes as well as the *mNSC-ablated* females – were also homozygous for the w^{1118} mutation, but the constructs contain a *mini-white* gene of which copies additively provide eye pigment. For this reason, UAS-*rpr* and GAL4-dilp3 heterozygote females had pale-orange eyes and the *mNSC*-ablated females orangey-red eyes (but still not wild-type in appearance). Focal males in all treatments possessed the wild-type dominant *white* gene (which gives red eyes) on the X chromosome, and were also homozygous for the recessive $sparkling^{poliert}$ mutation (*spa*). The female offspring of focal males therefore inherited an X-chromosome bearing the wild-type *white* gene from their father, and displayed wild-type red eyes, whereas the daughters of rival (w^{1118}) males were white or orange-eyed in the controls. Note that it is only possible to assign paternity for the focal male through the eye phenotype of daughters, sons could not be differentiated because they received the focal male's Y chromosome, not wild-type *white*-bearing X chromosome. Technical note: Although the figure represents the offspring of the crosses between UAS-*rpr* and GAL4-dilp3 heterozygote females and *white* rival males as having orange eyes, the offspring eye colour for these crosses could vary through white, pale orange or orangey-red depending on the presence of 0, 1 or 2 mini-white transgenes respectively. However, in all cases the eye colour was

clearly distinguishable from wild-type (i.e. the eye colour of focal male offspring).

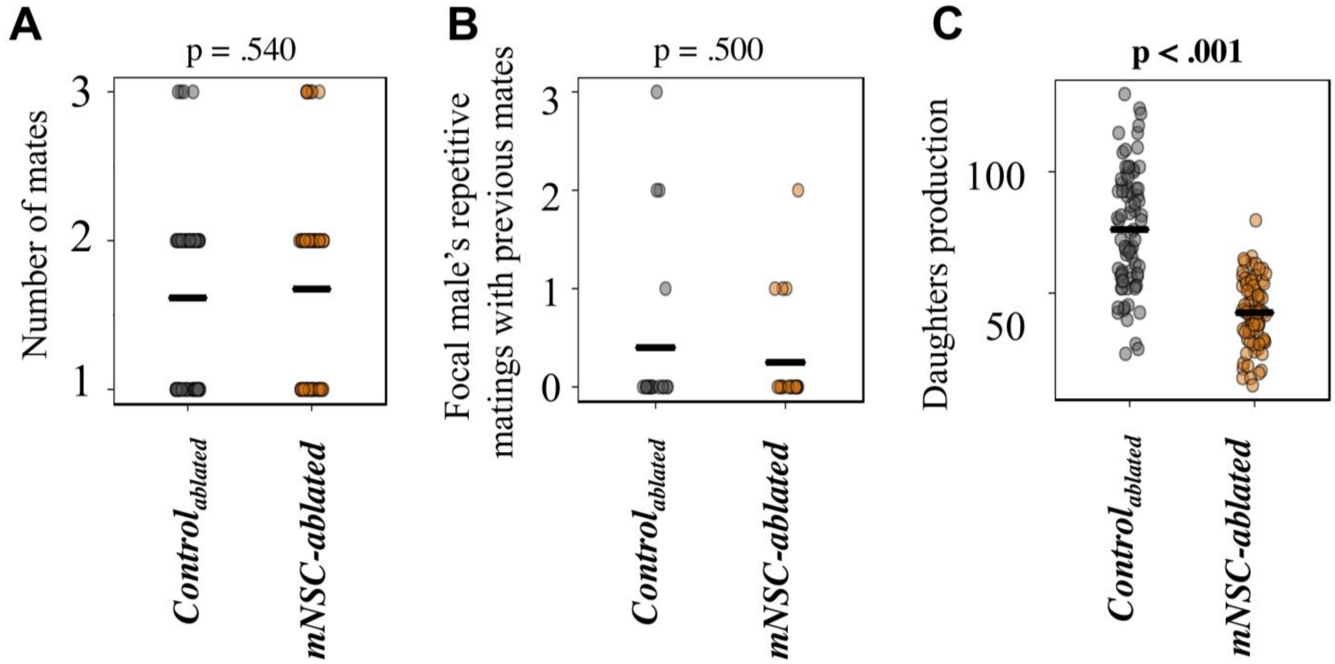

Supplementary Figure 5. Female *mNSC*-ablated mating and offspring data. **a.** The number of mates of females in the *mNSC-ablated* experiment. $N = 78$ for control$_{ablated}$ and $N = 80$ for mNSC-ablated. **b.** Focal male's number of matings with the same female. $N = 16$ for control$_{ablated}$ and $N = 16$ for mNSC-ablated. **c.** Offspring (i.e. daughter) production of females in the *mNSC-ablated* experiment. $N = 78$ for control$_{ablated}$ and $N = 80$ for mNSC-ablated. Grey refers to the control$_{ablated}$ treatment; Orange refers to the mNSC-ablated treatment. Horizontal black line represents the mean of the data.

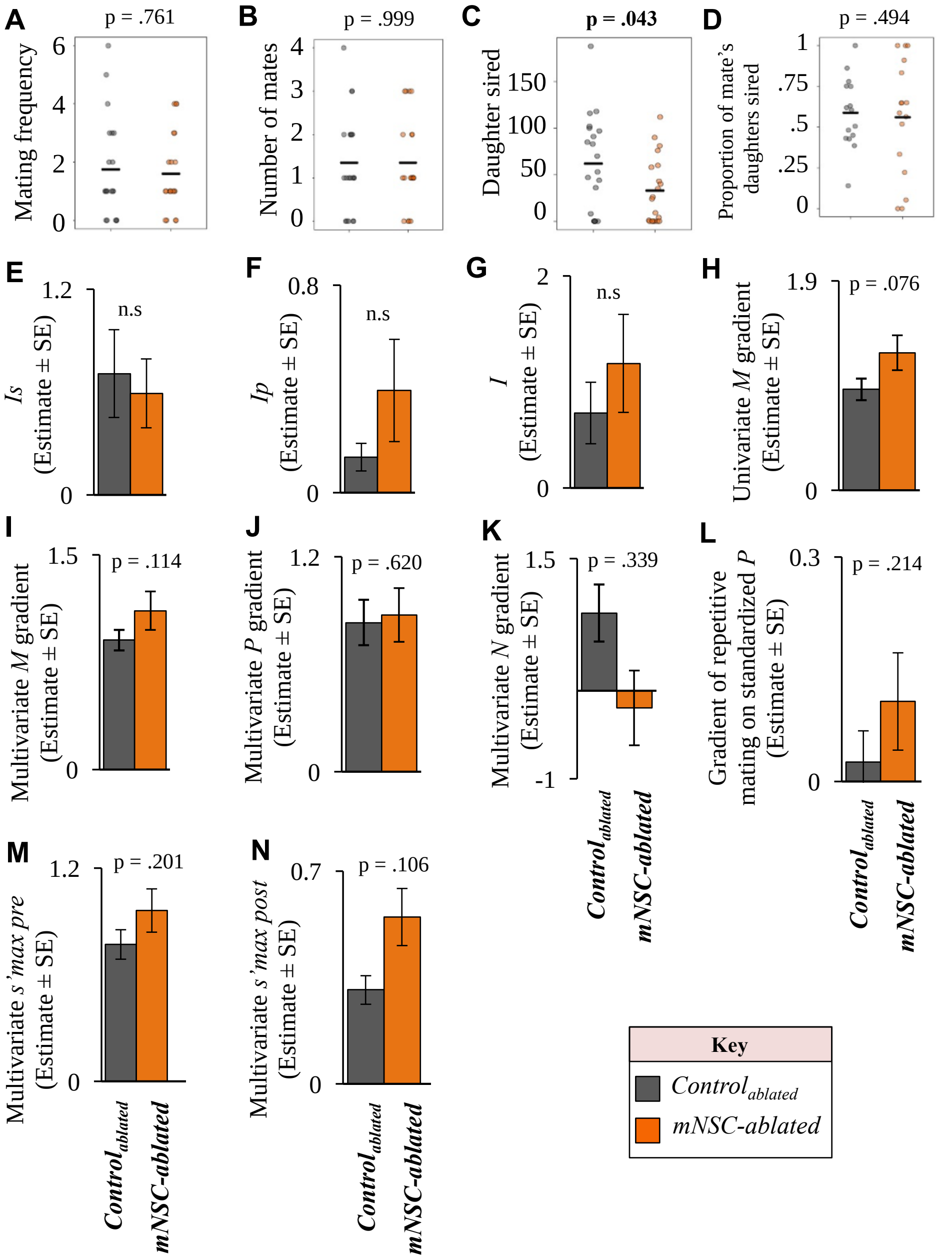

Supplementary Figure 6. Effects of reduced female productivity, via ablation of the mNSCs, on the operation of sexual selection in males. **a.** Focal male mating frequency (number of matings); **b.** Focal male mating success (number of mates); **c.** Number of daughters sired by the focal males; **d.** Proportion of daughters sired by the focal males with females they mated with; **e.** the standardized variance in focal male mating success (I_S), n.s: overlapping bootstrap confidence intervals; **f.** the standardized variance in focal male siring success (I_P) ("the opportunity for post-copulatory sexual selection"), n.s: overlapping bootstrap confidence intervals; **g.** the standardized variance in offspring (daughters) sired by the focal males (I) ("the opportunity for selection") n.s: overlapping bootstrap confidence intervals; **h.** The univariate gradient of focal male mating success and offspring (the univariate M gradient or "the Bateman gradient"); **i.** The multivariate gradient of focal male mating success and offspring (the multivariate M gradient); **j.** The multivariate gradient of focal male paternity share and offspring (the multivariate P gradient); **k.** The multivariate gradient of focal male mate productivity and offspring (the multivariate N gradient); **l.** the Gradient of repetitive matings with the same female. **m.** the maximum standardized multivariate pre-copulatory sexual selection differential index $s'_{max\,(pre)}$; **n.** the maximum standardized multivariate post-copulatory sexual selection differential index $s'_{max\,(post)}$. Panels **a** to **d**: Horizontal black line represents the mean of the data; p-values were obtained from F-tests from GLM models. Panels **e** to **n**: Error bars in barplots refer to the standard error of the mean (SE). Estimate – the estimate of the gradients. Grey bars refer to the control$_{ablated}$ treatment; Orange bars refer to the mNSC-ablated treatment. N = *16* for control$_{ablated}$ and N = *16* for mNSC-ablated.

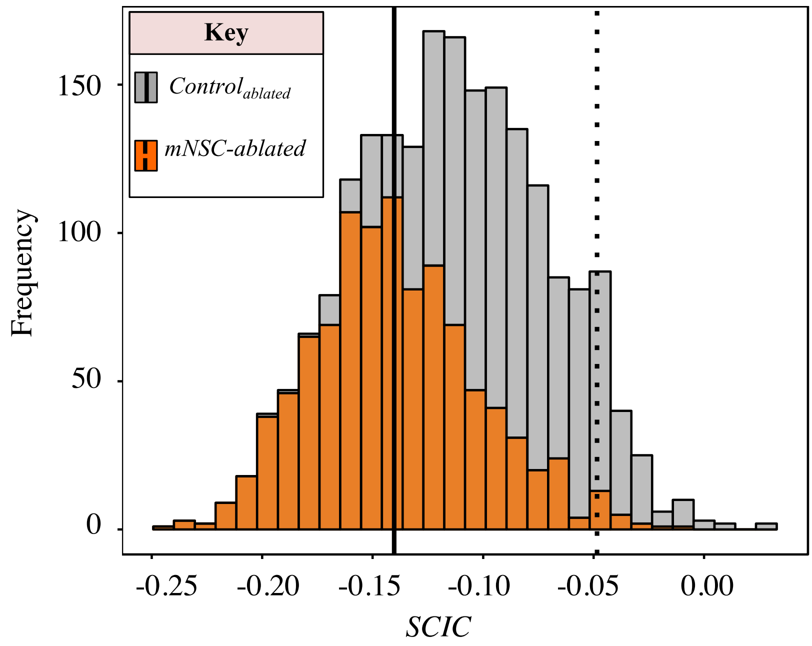

Supplementary Figure 7. Randomisations reveal no assortative mating patterns in populations where females have low productivity populations or control populations. Stacked frequency plot to show the simulated null distributions of sperm competition intensity correlation (*SCIC*) values generated from 1000 randomisations of empirical mating data for *mNSC-ablated* and control populations. Vertical lines highlight the observed *SCIC* value for each population. Observed values do not lie outside the range of values expected under the null hypothesis of no assortative mating (*Treatment: SCIC ± SE; control$_{ablated}$: -0.048 ± 0.077, p = 0.252; mNSC-ablated: -0.140 ± 0.077, p =0.937*). Grey bars refer to the control$_{ablated}$ treatment; Orange bars refer to the mNSC-ablated treatment.

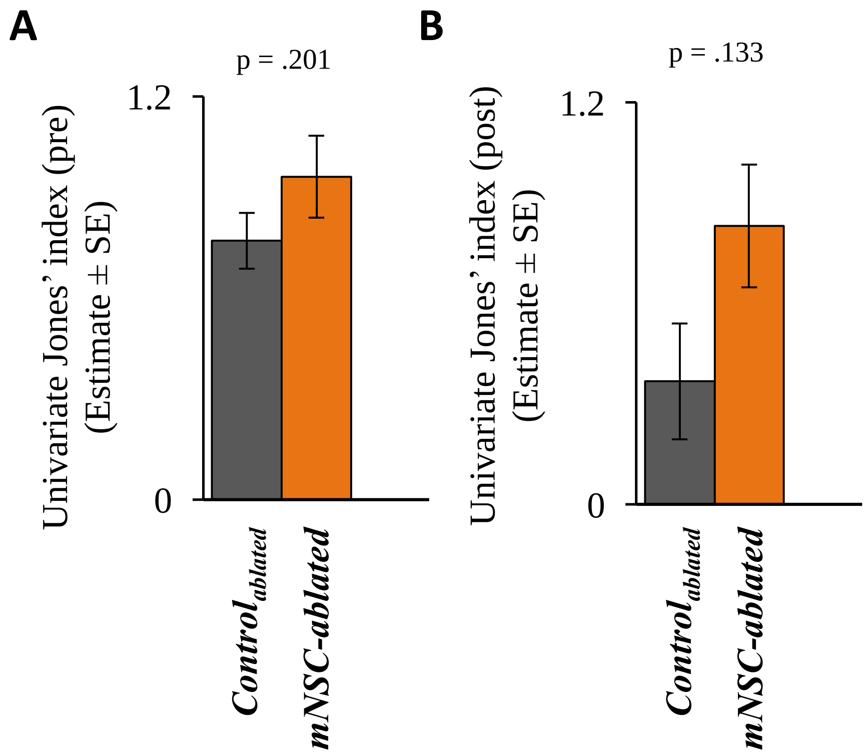

Supplementary Figure 8. The pre- and post-copulatory univariate Jones' Indexes in the mNSC-ablated experiment. **a.** The univariate pre-copulatory Jones' index. **b.** The univariate post-copulatory Jones' index. N = 16 for control-ablated and N = 16 for mNSC-ablated. Error bars refer to the standard error of the mean (SE). Grey bars refer to the $\text{control}_{\text{ablated}}$ treatment; Orange bars refer to the mNSC-ablated treatment.

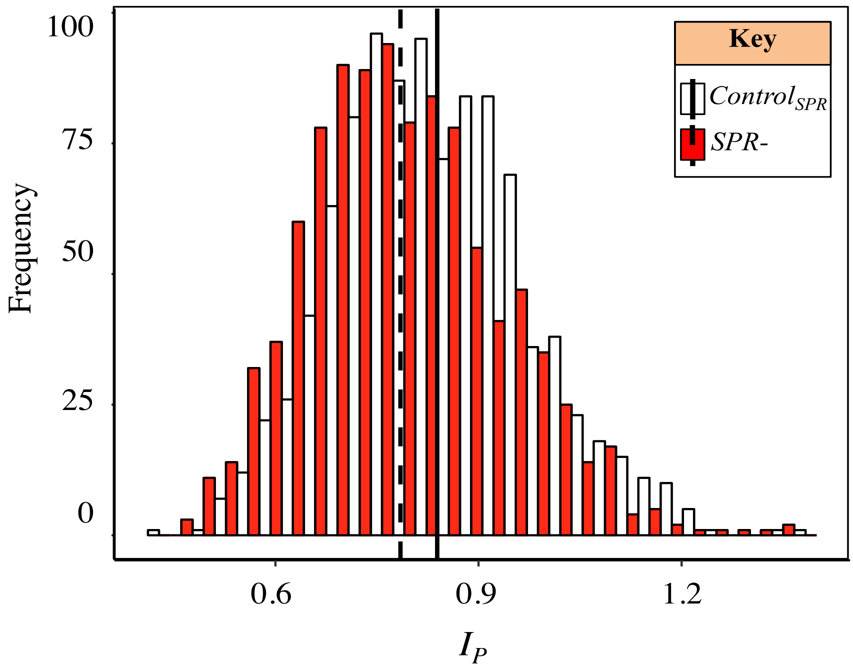

Supplementary Figure 9. Simulations reveal no evidence for female SPR effects on the opportunity for post-copulatory sexual selection in males. Frequency plot showing the simulated distributions of I_P when males mate with control$_{SPR}$ (white) and *SPR*-lacking (red) females. Values generated from 1000 simulations from empirical data from Smith et al. [2]. Vertical lines highlight the averages I_P in control$_{SPR}$ (solid) and *SPR*-lacking (dashed) females.

Supplementary Tables

Supplementary Table 1. Matings obtained by focal and non-focal (rival) males in control$_{SPR}$ and *SPR*- groups. SE refers to the standard error of the mean. *N* refers to the sample size.

**Treatment**	**Mean total matings per group**	**Male status**	**Mean total matings per male type**	**Mean matings per individual male type**	
				**Estimate (sample size)**	**SE**
Control$_{SPR}$	8.44	Focal	2.41	2.41 (N = 27)	0.303
		Non-focal	6.03	2.01 (N = 72)	0.116
SPR-	20.91	Focal	6.41	6.41 (N = 29)	0.402
		Non-focal	14.50	4.85 (N = 78)	0.289
Control$_{ablated}$	9.49	Focal	2.25	2.25 (N = 16)	0.371
		Non-focal	7.24	2.41 (N = 51)	0.161
mNSC-ablated	8.46	Focal	2.19	2.19 (N = 16)	0.319
		Non-Focal	6.27	2.09 (N = 55)	0.125

Supplementary Table 2. The opportunity for selection (*I*), pre-copulatory sexual selection (*Is*) and post-copulatory sexual selection (*Ip*). 95% bootstrap confidence intervals (CI).

**Index**	**Treatment**	**Estimate**	**Lower 95% CI**	**Upper 95% CI**
I	$Control_{SPR}$	0.591	0.372	1.083
	SPR-	0.386	0.259	0.601
	$Control_{ablated}$	0.707	0.348	1.602
	mNSC-ablated	1.175	0.599	2.589
I_S	$Control_{SPR}$	0.269	0.189	0.392
	SPR-	0.058	0.032	0.126
	$Control_{ablated}$	0.708	0.376	1.535
	mNSC-ablated	0.593	0.317	1.247
I_P	$Control_{SPR}$	0.263	0.156	0.494
	SPR-	0.270	0.189	0.489
	$Control_{ablated}$	0.137	0.075	0.448
	mNSC-ablated	0.394	0.162	1.083

Supplementary Table 3. Mean standardized univariate ('Bateman') and multivariate selection gradients. $\boldsymbol{\beta_{SS}^{Uni}}$ = Univariate mating success (*M)* gradient; β_{SS}^{Multi} = Multivariate mating success (*M)* gradient; β_{P}^{Multi} = Multivariate paternity (*P)* gradient; β_{N}^{Multi} = multivariate partner productivity (*N*) gradient; *T* = Treatment. **Bold** represents factors with p <0.05. *N* refers to the sample size. SE refers to the standard error of the mean.

**Factor**	**Control$_{SPR}$ (N = 27)**			**SPR- (i.e. high polyandrous) (N = 29)**			**Control$_{ablated}$ (N = 16)**			**mNSC-ablated (i.e. low productivity) (N = 16)**		
	Estimate	SE	p-value	Estimate	SE	p-value	Estimate	SE	p-value	Estimate	SE	p-value
β_{SS}^{Uni}	0.563	0.162	0.002	0.676	0.372	0.081	0.915	0.098	<0.001	1.248	0.158	<0.001
β_{SS}^{Multi}	0.720	0.101	<0.001	0.722	0.124	<0.001	0.904	0.072	<0.001	1.110	0.134	<0.001
β_{P}^{Multi}	0.618	0.097	<0.001	0.853	0.060	<0.001	0.833	0.127	0.001	0.875	0.150	<0.001
β_{N}^{Multi}	0.960	0.358	0.013	0.520	0.268	0.065	0.882	0.324	0.021	-0.195	0.424	0.654

**Slope comparison (N = 56)**				**Slope comparison (N = 32)**			
	Estimate	SE	p-value		Estimate	SE	p-value
$\beta_{SS}^{Uni} * T$	0.282	0.404	0.488	$\beta_{SS}^{Uni} * T$	0.330	0.180	0.076
$\beta_{SS}^{Multi} * T$	-0.015	0.180	0.933	$\beta_{SS}^{Multi} * T$	0.255	0.155	0.114
$\beta_{P}^{Multi} * T$	0.362	0.129	0.007	$\beta_{P}^{Multi} * T$	-0.104	0.208	0.620
$\beta_{N}^{Multi} * T$	0.228	0.111	0.045	$\beta_{N}^{Multi} * T$	-0.450	0.461	0.339

Supplementary Table 4. Variance standardized univariate and multivariate selection gradients. β_{SS}^{Uni} = Univariate mating success (*M)* gradient (pre-copulatory Jone's index); β_{SS}^{Multi} = Multivariate mating success (*M)* gradient; β_{P}^{Multi} = Multivariate paternity (*P)* gradient; β_{N}^{Multi} = multivariate partner productivity (*N*) gradient; *T* = Treatment. **Bold** represents factors with p <0.05. *N* refers to the sample size. SE refers to the standard error of the mean.

**Factor**	**Control$_{SPR}$ (*N* = 27)**			**SPR- (*N* = 29)**			**Control$_{ablated}$ (*N* = 16)**			**mNSC-ablated (*N* = 16)**		
	Estimate	SE	p-value	Estimate	SE	p-value	Estimate	SE	p-value	Estimate	SE	p-value
β_{SS}^{Uni}	0.340	0.091	0.035	0.162	0.089	0.336	0.77	0.083	<0.001	0.961	0.122	<0.001
β_{SS}^{Multi}	0.435	0.061	<0.001	0.174	0.029	<0.001	0.761	0.061	<0.001	0.855	0.103	<0.001
β_{P}^{Multi}	0.317	0.050	<0.001	0.443	0.031	<0.001	0.309	0.047	<0.001	0.549	0.094	<0.001
β_{N}^{Multi}	0.337	0.125	0.013	0.130	0.067	0.065	0.206	0.075	0.021	-0.051	0.111	0.654

**Slope comparison (N = 56)**				**Slope comparison (*N* = 32)**			
	Estimate	SE	p-value		Estimate	SE	p-value
$\beta_{SS}^{Uni} * T$	-0.100	0.133	0.452	$\beta_{SS}^{Uni} * T$	0.189	0.145	0.201
$\beta_{SS}^{Multi} * T$	-0.270	0.062	<0.001	$\beta_{SS}^{Multi} * T$	0.134	0.124	0.293
$\beta_{P}^{Multi} * T$	0.122	0.057	0.038	$\beta_{P}^{Multi} * T$	0.161	0.095	0.106
$\beta_{N}^{Multi} * T$	-0.283	0.077	<0.001	$\beta_{N}^{Multi} * T$	-0.102	0.112	0.373

Supplementary Table 5. The relationship between repetitive matings with the same female and fertilization success (*P*). **Bold** represents factors with p <0.05. *N* refers to the sample size. SE refers to the standard error of the mean.

**Factor**	**Control$_{SPR}$ (*N* = 27)**			**SPR- (*N* = 29)**			**Control$_{ablated}$ (*N* = 16)**			**mNSC-ablated (*N* = 16)**		
	Estimate	SE	p-value	Estimate	SE	p-value	Estimate	SE	p-value	Estimate	SE	p-value
Repetitive mating	-0.003	0.060	0.955	0.512	0.155	**0.002**	0.026	0.0417		0.536	0.107	0.065
Replicate	0.045	0.252	0.858	0.297	0.194	0.143	-	-	-	-	-	-
**Slope Comparison (*N* = 56)**							**Slope Comparison (*N* = 32)**					
	Estimate	SE	p-value				Estimate	SE	p-value			
Repetitive mating *Treatment	0.518	0.180	**0.006**				0.274	0.189	0.159			

Supplementary Table 6. The relationship between repetitive matings with the same female and adjusted paternity share (adjusted *PCS*) according to Devigili et al. [3]. *N* refers to the sample size. SE refers to the standard error of the mean.

**Factor**	**Control$_{SPR}$ (*N* = 27)**			**SPR- (*N* = 29)**			**Control$_{ablated}$ (*N* = 16)**			**mNSC-ablated (*N* = 16)**		
	Estimate	SE	p-value	Estimate	SE	p-value	Estimate	SE	p-value	Estimate	SE	p-value
Repetitive mating	-0.008	0.033	0.783	0.107	0.062	0.096	-0.024	0.029	0.421	0.057	0.410	0.187
Replicate	-0.125	0.141	0.385	0.149	0.077	0.066	-	-	-	-	-	-
**Slope Comparison (*N* = 56)**							**Slope Comparison (*N* = 32)**					
	Estimate	SE	p-value				Estimate	SE	p-value			
Repetitive mating * Treatment	0.103	0.083	0.222				0.085	0.048	0.094			

Supplementary Table 7. Decomposition of variance in male reproductive success (T) into the relative contributions of *M*, *P* and *N* in the low productivity experiment (mNSC-ablated). Delta method of variance decomposition following Webster et al. [4], Collet et al. [5], and Morimoto et al. [6].

**Var-Cov Components**	**Observed contribution to *var(T)***			
	**Control$_{ablated}$**		**mNSC-ablated**	
var(T)	1.854	%	1.255	%
var(M)	2.036	109.7	0.320	32.0
var(P)	1.025	55.2	0.470	47.1
var(N)	0.407	21.9	0.082	8.2
cov(M, P)	0.132	7.1	0.291	29.1
cov(M, N)	-0.643	34.6	0.177	17.7
cov(N, P)	0.052	2.8	0.174	17.4
D	-1.155		-0.259	

Supplementary Table 8. Comparison of reproductive phenotypes between UAS-rpr and GAL4-dilp3. We tested for differences in mating and reproductive success, proportion of offspring (daughters) sired by the focal male with females they mated with, between the heterozygotes *UAS-rpr* and *GAL4-dilp3*. Because there were no differences between the heterozygotes, we considered both as "*Controls*" in the *mNSC-ablated* experiment (see above).

	**Offspring**			**Mating frequency**		**Number of mates**		**Proportion of offspring sired**		**Average mate productivity of focal males**	
	df	F-value	p-value	F-value	p-value	F-value	p-value	F-value	p-value	F-value	p-value
Genetic construct (GAL4-*dilp3* vs. UAS-*rpr*)	1	1.166	0.295	0.255	0.619	0.600	0.449	0.337	0.572	0.155	0.700

Supplementary References

1. Yapici, N., et al., *A receptor that mediates the post-mating switch in Drosophila reproductive behaviour.* Nature, 2008. **451**(7174): p. 33-U1.
2. Smith, D. T., Clarke, N. V., Boone, J. M., Fricke, C. & Chapman, T. Sexual conflict over remating interval is modulated by the sex peptide pathway. *Proc R Soc Lond B Biol Sci* **284**, doi:10.1098/rspb.2016.2394 (2017)
3. Devigili, A., Evans, J. P., Di Nisio, A. & Pilastro, A. Multivariate selection drives concordant patterns of pre-and postcopulatory sexual selection in a livebearing fish. *Nat Commun* **6**, 8291, doi:10.1038/ncomms9291 (2015).
4. Webster, M. S., Pruett-Jones, S., Westneat, D. F. & Arnold, S. J. Measuring the effects of pairing success, extra-pair copulations and mate quality on the opportunity for sexual selection. *Evolution* **49**, 1147-1157, doi:10.2307/2410439 (1995).
5. Collet, J., Richardson, D. S., Worley, K. & Pizzari, T. Sexual selection and the differential effect of polyandry. *P Natl Acad Sci USA* **109**, 8641-8645, doi: 10.1073/Pnas.1200219109 (2012).
6. Morimoto, J., Pizzari, T. & Wigby, S. Developmental environment effects on sexual selection in male and female *Drosophila melanogaster*. *PLoS ONE* **11**, e0154468, doi:10.1371/journal.pone.0154468 (2016).
