# Supplementary material for: Sex peptide receptor-regulated polyandry modulates the balance of pre- and post-copulatory sexual selection in Drosophila
Source: Nat Commun. 2019 Jan 17;10:283. doi: 10.1038/s41467-018-08113-w (PMC6336784; doi:10.1038/s41467-018-08113-w)
Supplement: Supplementary file 3 — Reporting Summary [file 41467_2018_8113_MOESM3_ESM.pdf]

nature research

Corresponding author(s): Juliano Morimoto

# Reporting Summary

Nature Research wishes to improve the reproducibility of the work that we publish. This form provides structure for consistency and transparency in reporting. For further information on Nature Research policies, see Authors & Referees and the Editorial Policy Checklist.

## Statistical parameters

When statistical analyses are reported, confirm that the following items are present in the relevant location (e.g. figure legend, table legend, main text, or Methods section).

| n/a | Confirmed | |
|---|---|---|
| ☐ | ☒ | The exact sample size (*n*) for each experimental group/condition, given as a discrete number and unit of measurement |
| ☐ | ☒ | An indication of whether measurements were taken from distinct samples or whether the same sample was measured repeatedly |
| ☐ | ☒ | The statistical test(s) used AND whether they are one- or two-sided<br>*Only common tests should be described solely by name; describe more complex techniques in the Methods section.* |
| ☐ | ☒ | A description of all covariates tested |
| ☐ | ☒ | A description of any assumptions or corrections, such as tests of normality and adjustment for multiple comparisons |
| ☐ | ☒ | A full description of the statistics including central tendency (e.g. means) or other basic estimates (e.g. regression coefficient) AND variation (e.g. standard deviation) or associated estimates of uncertainty (e.g. confidence intervals) |
| ☐ | ☒ | For null hypothesis testing, the test statistic (e.g. *F*, *t*, *r*) with confidence intervals, effect sizes, degrees of freedom and *P* value noted<br>*Give P values as exact values whenever suitable.* |
| ☒ | ☐ | For Bayesian analysis, information on the choice of priors and Markov chain Monte Carlo settings |
| ☒ | ☐ | For hierarchical and complex designs, identification of the appropriate level for tests and full reporting of outcomes |
| ☒ | ☐ | Estimates of effect sizes (e.g. Cohen's *d*, Pearson's *r*), indicating how they were calculated |
| ☐ | ☒ | Clearly defined error bars<br>*State explicitly what error bars represent (e.g. SD, SE, CI)* |

*Our web collection on statistics for biologists may be useful.*

## Software and code

Policy information about availability of computer code

| | |
|---|---|
| Data collection | R Statistical Software |
| Data analysis | R Statistical Software |

For manuscripts utilizing custom algorithms or software that are central to the research but not yet described in published literature, software must be made available to editors/reviewers upon request. We strongly encourage code deposition in a community repository (e.g. GitHub). See the Nature Research guidelines for submitting code & software for further information.

## Data

Policy information about availability of data

All manuscripts must include a data availability statement. This statement should provide the following information, where applicable:

- Accession codes, unique identifiers, or web links for publicly available datasets
- A list of figures that have associated raw data
- A description of any restrictions on data availability

Data is available in the Oxford Research Archive (ORA) [(https://doi.org/10.5287/bodleian:J5kpxjJB0)]

# Field-specific reporting

Please select the best fit for your research. If you are not sure, read the appropriate sections before making your selection.

☐ Life sciences ☐ Behavioural & social sciences ☒ Ecological, evolutionary & environmental sciences

For a reference copy of the document with all sections, see nature.com/authors/policies/ReportingSummary-flat.pdf

# Ecological, evolutionary & environmental sciences study design

All studies must disclose on these points even when the disclosure is negative.

| | |
|---|---|
| Study description | Groups of 4 males and 4 females flies of varying polyandry levels. Individual behaviour in replicate groups was assessed. Reproduction was assessed for all females and a focal male. |
| Research sample | Groups of individually maked individuals. Offspring number and paternity was assessed. |
| Sampling strategy | Behavioural observation. |
| Data collection | Juliano Morimoto, Grant C McDonald, Emelia Smith, Damian Smith collected data. Matings were scored individually. Offspring was counted, and paternity share of each daughter was assessed through phenotypic markers. |
| Timing and spatial scale | Data was collected for 4 consecutive days. 4 hours of behavioural interactions, 20h of oviposition and offspring production per day. |
| Data exclusions | N/A |
| Reproducibility | We repeated the experiments to verify our findings. The results were consistent between experimental replicates. |
| Randomization | This is not relevant. WE produced flies in extremely large numbers and therefore biases were unlikely to have been generated. All measures were taken to minimise biases, such as varying the paint colour that flies were marked with. |
| Blinding | Scientists observed mating behaviour blindly with respect to groups. Blinding was not possible for offspring data because the scientists needed to know the groups from which offspring came from in order to assess paternity via specific phenotypic markers. This was unlikely to generate biases because there are clear distinction on the phenotype of flies in the experimental and control groups. |

Did the study involve field work? ☐ Yes ☒ No

# Reporting for specific materials, systems and methods

## Materials & experimental systems

| n/a | Involved in the study |
|---|---|
| ☒ | ☐ Unique biological materials |
| ☒ | ☐ Antibodies |
| ☒ | ☐ Eukaryotic cell lines |
| ☒ | ☐ Palaeontology |
| ☐ | ☒ Animals and other organisms |
| ☒ | ☐ Human research participants |

## Methods

| n/a | Involved in the study |
|---|---|
| ☒ | ☐ ChIP-seq |
| ☒ | ☐ Flow cytometry |
| ☒ | ☐ MRI-based neuroimaging |

## Animals and other organisms

Policy information about studies involving animals; ARRIVE guidelines recommended for reporting animal research

| | |
|---|---|
| Laboratory animals | Drosophila melanogaster, SPR knockout, UAS-GAL4-DILP4 (mNSC-ablated) |
| Wild animals | N/A |
| Field-collected samples | N/A |
